# Angiogenesis in Glioblastoma—Treatment Approaches

**DOI:** 10.3390/cells14060407

**Published:** 2025-03-11

**Authors:** Agnieszka Nowacka, Maciej Śniegocki, Wojciech Smuczyński, Dominika Bożiłow, Ewa Ziółkowska

**Affiliations:** 1 Department of Neurosurgery, Nicolas Copernicus University in Toruń, Collegium Medicum in Bydgoszcz, ul. Curie Skłodowskiej 9, 85-094 Bydgoszcz, Poland; 2 Department of Physiotherapy, Nicolas Copernicus University in Toruń, Collegium Medicum in Bydgoszcz, ul. Techników 3, 85-801 Bydgoszcz, Poland; 3 Anaesthesiology and Intensive Care Clinical Ward, The 10th Military Research Hospital and Polyclinic, ul. Powstańców Warszawy 5, 85-681 Bydgoszcz, Poland; 4 Department of Pediatrics, Washington University School of Medicine, St. Louis, MO 63110, USA; eziolkowska@wustl.edu

**Keywords:** glioblastoma, GBM, angiogenesis, VEGF, bevacizumab, anti-angiogenic therapy, tumor microenvironment, resistance

## Abstract

Glioblastoma, the most common primary malignant brain tumor in adults, carries a poor prognosis, with a median survival of just 15 months, significantly impacting patients’ quality of life. The aggressive growth of these highly vascularized tumors relies heavily on angiogenesis, driven primarily by vascular endothelial growth factor-A. Therefore, VEGF signaling pathway has become a prime therapeutic target in GBM treatment over the past decade. While anti-angiogenic treatment showed promise, agents like bevacizumab have ultimately failed to improve overall survival. This highlights the presence of compensatory angiogenic mechanisms that bypass VEGF inhibition, necessitating further investigation into resistance mechanisms and the development of more effective therapeutic strategies. This review examined the current landscape of anti-angiogenic agents for GBM, analyzed the mechanisms driving resistance to these therapies, and explored potential strategies for enhancing their effectiveness.

## 1. Introduction

Glioblastoma (GBM) stands as one of the most aggressive and prevalent primary brain tumors originating from glial cells within the central nervous system (CNS) [1,2]. Classified as a high-grade glioma (grade IV glioma differentiating from astrocytic cells) [3], GBM is the most aggressive form, accounting for 54% of CNS gliomas in the US, with the annual incidence ranging from 3.19 to 4.17 per 100,000 person–years [4,5]. In the pediatric population (0–18 years), the incidence is lower (0.85 per 100,000), and pediatric GBM represents 3–15% of primary brain tumors [5]. GBM predominantly affects individuals aged 50–60, with a higher prevalence in men [6]. It occurs more frequently supratentorially, often in the frontal lobe, with the brainstem and cerebellum being the rarest locations [5]. GBM classification is based on IDH (isocitrate dehydrogenase) status, a distinction introduced in the 2016 WHO classification of CNS tumors, incorporating both histopathological and molecular criteria [5]. IDH wild-type GBM (90% of cases) peaks in incidence during the sixth decade of life, while IDH-mutant GBM (10%) occurs earlier, in the fourth and fifth decades, and carries a better prognosis [5]. The classification also includes GBM, NOS (not otherwise specified), and NEC (not elsewhere classified) for cases with unclear IDH status or those not fitting existing categories [5]. The 2021 WHO classification further emphasizes and refines the molecular characterization of brain tumors [7].

Brain tumor symptoms are broadly classified as general or focal. Small, low-grade tumors tend to cause focal symptoms, while larger, more advanced tumors present with generalized symptoms [8]. General symptoms include headache, vomiting, nausea, cognitive and emotional impairment, and sensory deficits [8]. Due to the often asymptomatic nature of early-stage GBM multiforme, diagnosis typically occurs at later stages [9]. GBM symptoms are non-specific and depend largely on tumor location but commonly include headache, nausea, visual disturbances, motor problems, seizures, personality changes, and significant memory impairment [9]. Patients with high-grade gliomas are also prone to depression and anxiety, impacting their well-being and quality of life [10]. Cancer-related fatigue is another common symptom [8]. Epilepsy, triggered by tumor growth and invasion along white matter fiber tracts, is also frequently observed in glioma patients [11]. Studies have shown that glioma-related epilepsy affects the white matter fiber microstructure within the tumor itself [11].

Rapid and accurate diagnosis is crucial for timely and effective treatment of brain tumors. Magnetic resonance imaging has become the gold standard for glioma evaluation, providing clear visualization of anatomical structures without the interference of skull artifacts [12]. MRI employs various modalities, including T1-weighted, T1-weighted contrast-enhanced, T2-weighted, and T2-weighted FLAIR, to differentiate between tissue types [12]. However, manual review of MRI scans is time-consuming and susceptible to human error. Consequently, there has been a surge in research exploring the application of artificial intelligence and deep learning to enhance diagnostic accuracy and efficiency, minimizing the risk of misdiagnosis [13,14,15].

GBM presents a formidable therapeutic challenge due to its high recurrence rate despite aggressive treatment [16]. It remains an incurable cancer with a median survival of approximately 15 months in treated patients and a 5-year survival rate of only 5%, influenced by age at diagnosis, molecular characteristics, extent of resection, and treatment response. The current standard of care involves maximal safe surgical resection followed by concurrent chemoradiation therapy [16]. This typically consists of 60 Gray of radiation delivered in 30 fractions over 6 weeks, combined with daily temozolomide administration, followed by adjuvant temozolomide [16]. However, treatment efficacy is limited by factors such as local tumor invasion and infiltration, the blood–brain barrier hindering chemotherapy penetration, and the development of multidrug resistance [17]. Consequently, novel therapeutic strategies are being actively explored, including immunotherapy approaches such as peptide vaccines, dendritic cell vaccines, chimeric antigen receptor T-cell therapy, checkpoint inhibitors, and oncolytic virotherapy [18,19,20]. While some of these methods have shown promise, significant challenges remain particularly immunosuppression within the tumor microenvironment [18].

A hallmark of GBMs is their highly vascular nature, with angiogenesis playing a central role in their growth and survival. Endothelial cells construct the tumor’s blood vessels, supplying essential oxygen and nutrients. Furthermore, these vessels directly promote the proliferation of GBM progenitor cells via intercellular signaling pathways, further enhancing tumor development and viability [21]. This intricate relationship between angiogenesis and tumor progression has become a focal point for therapeutic development, with researchers actively pursuing drugs that target these mechanisms to improve prognosis and long-term survival for brain tumor patients.

## 2. Angiogenesis in Glioblastoma

**Figure 1:**
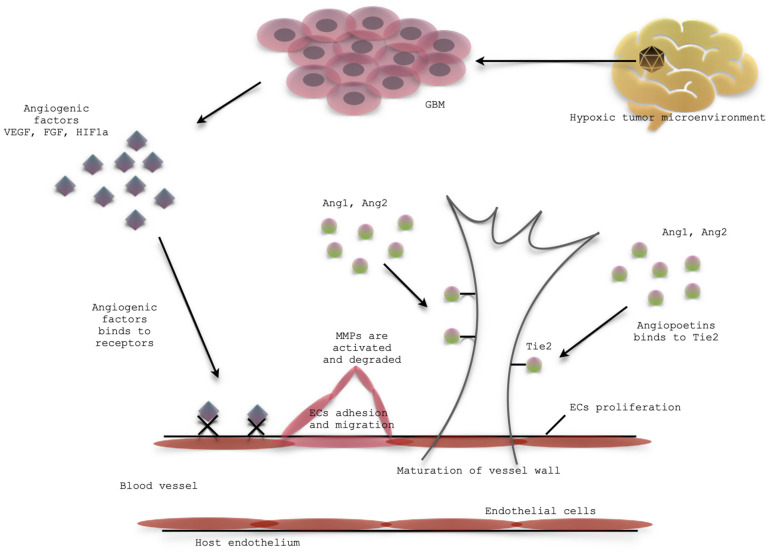
Glioblastoma angiogenesis.

Angiogenesis, the formation of new blood vessels from existing ones, plays a crucial role in both physiological and pathological processes [22]. This process is tightly regulated by a balance of pro- and anti-angiogenic factors. In GBM, angiogenesis occurs in the later stages of tumor development, primarily within the necrotic niche (Figure 1). Key angiogenic factors driving this process include hypoxia-inducible factor 1 (HIF-1α), vascular endothelial growth factor (VEGF), fibroblast growth factor (FGF), angiopoietin-1 (Ang-1), and angiopoietin-2 (Ang-2). Hypoxia serves as the primary trigger, activating HIF-1α, which in turn regulates the expression of growth factors [23], metabolic proteins [24], matrix components [25], and adhesion molecules [26]. Oxygen deprivation stimulates the release of VEGF [27], FGF [28], and angiopoietins [29], which bind to their respective receptors on endothelial cells. This binding initiates the dissolution of the vessel wall, degradation of the endothelial basement membrane (ECM) and extracellular matrix, and remodeling of the ECM by matrix metalloproteinases (MMPs). Subsequently, stromal cells synthesize new matrix components, promoting endothelial cell proliferation and migration, leading to the formation of tube-like endothelial structures [30]. The final stage involves the development of a mature vascular basement membrane around these newly formed structures, with pericytes and smooth muscle cells (mural cells) providing support and stability to the nascent vessels.

Tumor-associated macrophages (TAMs) play a significant role in GBM angiogenesis [31,32]. Constituting up to 30% of the tumor microenvironment, these macrophages originate from blood monocytes or activated microglia within brain tissue [32,33]. TAMs secrete a variety of inflammatory cytokines and growth factors, including TGF-β (Transforming Growth Factor-β), VEGF, IL-10 (Interleukin-10), and TNF-α (Tumor Necrosis Factor-α), which promote endothelial cell survival and proliferation, thereby supporting angiogenesis, tumor immunosuppression, and metastasis [31,32]. Additionally, TAMs indirectly contribute to tumor progression by activating other immune cells and remodeling the extracellular matrix [33]. Consequently, a high TAM presence correlates with increased tumor growth, poorer patient prognosis, and a greater risk of recurrence after treatment [34].

Angiogenesis is essential for tumor growth and progression. Tumors develop abnormal, immature blood vessels characterized by larger size, irregular paths, varying lumen diameters, high permeability, and erratic branching [35]. This abnormal vasculature is permeable to plasma and its proteins, leading to local edema and extravascular clotting [36]. Consequently, interstitial pressure increases, disrupting blood flow and leukocyte infiltration [37]. The flawed basement membrane and lack of proper perivascular connective tissue facilitate tumor cell dissemination [38]. Compression and leakiness within the tumor vasculature obstruct the delivery of oxygen, nutrients, and therapeutic drugs, resulting in ischemia, necrosis, and a hypoxic environment that further stimulates HIF-1 activation and angiogenesis [39,40,41]. This disordered vasculature profoundly alters the tumor microenvironment, influencing growth, metastasis, and treatment resistance. Therefore, targeting tumor vasculature and inhibiting associated growth factors and signaling pathways represent promising therapeutic strategies.

Vascular endothelial growth factors are essential regulators of blood and lymphatic vessel formation and function in both physiological and pathological contexts [42]. Within the central nervous system, VEGF plays a role in angiogenesis, neuronal migration, and neuroprotection. However, as a permeability factor, excessive VEGF levels can disrupt intracellular barriers, increase choroid plexus endothelial leakage, induce edema, and activate inflammatory pathways [43]. The VEGF gene, located at 6p21.3, belongs to the cysteine knot growth factor superfamily, which also includes PDGF, NGF, and TGF-β [44]. In mammals, the VEGF family comprises VEGF-A, VEGF-B, VEGF-C, VEGF-D, and placental growth factor, encoding structurally homologous glycoproteins [45]. These proteins form homodimers or heterodimers via cysteine disulfide bridges, a crucial step for their biological activity [46].

VEGF-A, the most extensively studied member of the VEGF family, exists in various isoforms, such as VEGF-A121, VEGF-A165, and VEGF-A189 [46,47]. These isoforms arise from alternative mRNA splicing, resulting in differences in bioavailability and biological potency [46]. Vascular endothelial growth factor-A represents the principal angiogenic factor expressed within the solid tumor microenvironment [44]. Tumor cells, in particular, secrete high concentrations of VEGF-A, driving cell proliferation, migration, and angiogenesis.

VEGF-B is highly expressed in the heart, particularly in cardiomyocytes, where it plays a crucial role in regulating myocardial contractility and protecting cardiomyocytes from ischemic and apoptotic damage, leading to physiological hypertrophy [48]. It is also involved in cardiac remodeling after myocardial infarction [48].

VEGF-C and VEGF-D are key players in lymphangiogenesis, promoting the proliferation of lymphatic endothelial cells [49]. VEGF-C, in particular, is implicated in promoting lymphangiogenesis in various cancers [50]. Furthermore, VEGF-D expression by cancer cells is known to facilitate metastasis [50].

Placental growth factor (PlGF), the final member of the VEGF family, stimulates the growth of endothelial and smooth muscle cells. In conjunction with VEGF-B, PlGF participates in monocyte differentiation and activation. Elevated PlGF concentrations have been observed in myocardial infarction and various cancers [49].

Vascular endothelial growth factor signaling is mediated by three tyrosine kinase receptors: VEGFR1 (vascular endothelial growth factor receptor 1, FLT1), VEGFR2 (vascular endothelial growth factor receptor 2, KDR/FLK1), and VEGFR3 (vascular endothelial growth factor receptor 3, FLT4) [46]. Each receptor possesses seven extracellular immunoglobulin-like domains and an intracellular tyrosine kinase domain activated upon VEGF binding. VEGFR1 exhibits a tenfold higher affinity for VEGF than VEGFR2, promoting endothelial and inflammatory cell migration during pathological angiogenesis [51]. VEGF binding to VEGFR2 activates pathways like PLCγ/PKC, contributing to both physiological and pathological angiogenesis, along with anti-apoptotic and cell migration effects. VEGFR3 primarily influences lymphangiogenesis in embryonic development and pathological conditions, including lymphatic metastasis. These receptors display varying affinities for different VEGF proteins and isoforms: VEGFR1 binds VEGF-A, VEGF-B, and PlGF; VEGFR2 binds VEGF-A and processed VEGF-C and VEGF-D; and VEGFR3 exhibits the strongest affinity for VEGF-C and VEGF-D [46,51]. Among these receptors, VEGFR2 is the primary mediator of VEGF signaling in endothelial cells, playing a crucial role in VEGF-induced endothelial functions [47,52].

Heparan sulfate proteoglycans and neuropilin 1 and 2 (NRP1/2) co-receptors are crucial for proper VEGF-VEGFR interactions [50,53]. NRPs, found on immune, cancer, and endothelial cells, enhance VEGF signaling and promote angiogenesis upon VEGF binding [53]. Interestingly, NRPs can also contribute to tumor formation independently of VEGF. HSPs interact with various signaling pathways and play a significant role in key steps of carcinogenesis, including tumor cell migration, anti-apoptotic activity, metastasis, and angiogenesis [54].

In malignant gliomas, VEGF-A is the most important glycoprotein secreted during angiogenesis, acting as a central player in tumor biology [55]. High VEGF expression is observed in necrotic areas of GBM, induced by HIF-1α under hypoxic conditions [56,57]. GBM formation involves the induction of VEGFR-1 in endothelial cells, while malignant progression requires the coordinated function of both VEGFR-1 and VEGFR-2 [58]. Upon binding to its receptors on endothelial cells, VEGF triggers the secretion of matrix metalloproteinases, which degrade the extracellular matrix, facilitating endothelial cell migration and proliferation [59]. Due to its significant role in tumor development, VEGF and its receptors have become key targets for anti-cancer therapy.

## 3. Anti-Angiogenic Therapy

Given the highly vascularized nature of gliomas, targeting angiogenesis seems to represent an effective treatment strategy. Numerous anti-angiogenic agents (Table 1 and Table 2) are either currently used in GBM treatment or are approved for other malignancies and are being explored for their potential use in GBM.

### 3.1. Aflibercept

Aflibercept, a recombinant fusion protein designed to bind and neutralize vascular endothelial growth factor and placental growth factor, key players in tumor angiogenesis, has emerged as a potential treatment strategy for GBM [89]. Studies indicate that aflibercept administration leads to a substantial decrease in circulating VEGF levels within 24 h, a reduction that correlates with positive radiographic responses, particularly in patients with recurrent GBM [89,90]. This suggests a direct link between VEGF suppression and tumor response. Furthermore, research has identified potential biomarkers for predicting aflibercept efficacy. Patients who respond well to aflibercept tend to exhibit elevated baseline levels of specific chemokines, including CTACK/CCL27 (cutaneous T-cell-attracting chemokine/chemokine C-Cmotif ligand 27), MCP3/CCL7 (monocyte-chemotactic protein 3/chemokine C-Cmotif ligand 7), MIF (macrophage migration inhibitory factor), and IP-10/CXCL10 (interferon gamma-induced protein 10/C-X-C Motif Chemokine Ligand 10) [89,90]. Additionally, a decrease in VEGFR1+ monocytes following treatment is associated with improved patient outcomes [89,90]. These findings highlight the potential for personalized treatment approaches based on individual biomarker profiles.

The clinical application of aflibercept is not without challenges. Its safety and toxicity profile are complex, requiring careful consideration. When administered in combination with radiation and temozolomide, the maximum tolerated dose of aflibercept is 4 mg/kg every two weeks [91]. Dose-limiting toxicities, including deep vein thrombosis and neutropenia, have been observed at higher doses. Moreover, changes in cytokine levels, such as IL-6 (interleukin 6), IL-10 (interleukin 10), and IL-13 (interleukin 13), are associated with treatment-related toxicities, notably fatigue and endothelial dysfunction [63]. These adverse effects can be significant and often lead to treatment discontinuation, underscoring the need for close patient monitoring and management of potential side effects.

While aflibercept holds promise for managing GBM, its use is complicated by the potential for significant toxicities and the need for careful patient monitoring. Identifying and validating biomarkers for both efficacy and toxicity could pave the way for more personalized treatment strategies, potentially improving outcomes and minimizing adverse effects.

### 3.2. Axitinib

Axitinib, a VEGFR tyrosine kinase inhibitor, has shown promise in preclinical GBM models, particularly by targeting GBM stem-like cells and tumor vasculature [92,93].

In preclinical animal studies, high-dose axitinib treatment effectively reduced tumor blood vessel density, increased immune cell infiltration, and caused significant tumor cell death [93]. These anti-tumor effects were linked to axitinib’s inhibition of the PDGFR/ERK signaling pathway, which plays a crucial role in tumor growth and survival [93].

Despite the preclinical promise, axitinib’s efficacy in GBM remains limited by factors like blood–brain barrier penetration and tumor heterogeneity [92]. Future research should focus on optimizing combinations and identifying biomarkers for patient selection.

### 3.3. Bevacizumab

Bevacizumab (BEV) is a recombinant humanized monoclonal antibody that specifically targets vascular endothelial growth factor, a key regulator of angiogenesis [94]. Classified as an anti-angiogenic agent, bevacizumab inhibits the formation of new blood vessels, a process crucial for tumor growth and metastasis. Its mechanism of action involves binding to circulating VEGF, thereby preventing its interaction with VEGF receptors on the surface of endothelial cells [94]. This blockade disrupts the signaling cascade that normally leads to endothelial cell proliferation, migration, and the formation of new blood vessels. Consequently, tumor growth is suppressed due to the deprivation of oxygen and nutrients supplied by the newly formed vasculature. Bevacizumab is clinically used in the treatment of various cancers, including GBM, colorectal cancer, non-small cell lung cancer, and renal cell carcinoma [95]. In GBM, bevacizumab targets the abnormal tumor vasculature, although its efficacy can be variable.

Studies have revealed a correlation between VEGFA expression levels in GBM tissues and treatment response to bevacizumab. Specifically, high VEGFA expression has been associated with improved progression-free survival after bevacizumab treatment [96]. Statistical analyses have demonstrated a significant difference in PFS (Progression-Free Survival) between patients with high and low VEGF-A expression, with high expressers experiencing a PFS of 10 months compared to 4 months for low expressers [96]. This suggests that patients with higher VEGF-A levels may derive greater benefit from bevacizumab therapy, experiencing longer periods without disease progression. Consequently, VEGF-A holds promise as a potential biomarker for predicting bevacizumab treatment response, enabling personalized treatment strategies and potentially improving outcomes for GBM patients.

Melhem et al. investigated the efficacy of low-dose versus standard-dose bevacizumab in recurrent GBM (rGBM) patients. The LD (low dose) regimen (5 mg/kg every 2–3 weeks or 10 mg/kg every 3 weeks) demonstrated significantly improved outcomes compared with the SD (standard dose) regimen (10 mg/kg every 2 weeks) [97]. Patients receiving LD BEV experienced a longer median progression-free survival (5.89 vs. 3.22 months) and overall survival (10.23 vs. 6.28 months) [97]. The LD regimen was associated with a 2.67-fold reduction in disease progression likelihood and a 2.56-fold improvement in survival chances [97]. Notably, the LD group had a higher median age (62 vs. 54 years) yet still exhibited superior outcomes [97]. While adverse events like fatigue, arthralgia, and hypertension were more common in the LD group, serious adverse events leading to treatment discontinuation were rare [97]. These findings suggest that LD BEV offers a survival benefit and potential cost-effectiveness, potentially broadening treatment access.

A study analyzing data from 106 GBM IDH-wildtype patients, 39 of whom received bevacizumab as a second-line treatment, found a significant difference in median survival from tumor progression based on tumor vascularity [98]. Patients with moderate vascular tumors treated with bevacizumab lived a median of 305 days longer than those without second-line treatment, while those with high vascular tumors lived only 173 days longer [98]. Patients with moderate vascularity showed better responses to bevacizumab, with a higher proportion surviving at 6, 12, 18, and 24 months post-progression [98]. The study proposes rCBV (relative Cerebral Blood Volume) max, with a threshold of 7.5, as a predictive biomarker for bevacizumab benefit, potentially improving personalized treatment decisions.

The efficacy of bevacizumab is limited, necessitating further therapeutic exploration. The latest research by Lai et al. has shown a correlation between bevacizumab and ROCK2 (Rho-associated coiled-coil forming kinase-2). Rho-associated kinase 2, a component of the Rho/ROCK (Rho-kinase/Rho-associated coiled-coil forming kinase) signaling pathway, regulates cellular processes like motility, migration, and proliferation, making it a potential therapeutic target in cancer [99]. Inhibiting ROCK2 has been shown to enhance bevacizumab’s effects in GBM by reducing GBM cell viability and migration, primarily through the RhoA/ROCK2 (Rho-kinase-A/Rho-associated coiled-coil forming kinase) pathway, leading to increased apoptosis [99]. Additionally, ROCK2 inhibition reduces angiogenesis and the degradation of cellular matrix-related cytokines, crucial processes in tumor growth and metastasis [99]. This synergistic effect of ROCK2 inhibition with bevacizumab presents a promising strategy for improving GBM treatment outcomes.

The latest meta-analysis of phase II and III randomized controlled trials indicated that bevacizumab significantly improves progression-free survival in GBM patients but does not prolong overall survival (OS) [67]. It is effective in both first-line and second-line treatments and shows improved PFS regardless of MGMT (O6-methylguanine-DNA methyltransferase) methylation status when combined with temozolomide [67]. However, the use of bevacizumab is associated with increased risks of hypertension, proteinuria, thromboembolic events, and infections, necessitating careful monitoring, particularly for hypertension [67]. Interestingly, the development of hypertension in GBM patients receiving bevacizumab has been associated with improved overall survival, suggesting a potential role as a biomarker for treatment response [95]. It is hypothesized that hypertension may influence the interplay between tumor cells and the perivascular niche, a specialized microenvironment surrounding blood vessels [95]. This altered interaction could potentially impact tumor invasion and growth dynamics. Increased blood pressure may reshape the perivascular microenvironment by modifying vascular permeability, blood flow, and the extracellular matrix composition. These alterations could consequently impact tumor cell migration, proliferation, and survival, ultimately influencing disease progression.

Bevacizumab’s primary clinical benefit, which contributes to its widespread use as the most commonly employed anti-angiogenic agent in the treatment of recurrent GBM, is its capacity to alleviate brain edema. It effectively relieves symptoms of radiation brain necrosis, improving the Karnofsky performance status and enhancing brain necrosis imaging [100]. By binding to VEGF and preventing its interaction with endothelial cell receptors, bevacizumab reduces vascular permeability and brain edema [100]. Its long half-life and convenient administration are advantageous. However, bevacizumab only addresses symptoms from new vessel formation around necrotic areas, and recurrence is possible due to the reactivation of the HIF-1α/VEGF cycle [100]. While recurrence due to excessive vessel pruning and ischemia has been observed, bevacizumab resistance in this context is not yet conclusively reported [100]. Further clinical data are needed to refine indications, optimize protocols, and address resistance and recurrence while emphasizing preventative strategies through careful radiotherapy dose management.

### 3.4. Cediranib

Cediranib, an oral pan-VEGF receptor tyrosine kinase inhibitor, has shown promise in treating GBM, particularly in newly diagnosed cases [101]. Its mechanism of action involves normalizing tumor vasculature, reducing endothelial proliferation, and maintaining blood–brain barrier integrity [102]. A study comparing recurrent GBMs (rGBM) treated with cediranib to those without anti-angiogenic therapy revealed several key findings [102]. Cediranib treatment led to decreased endothelial proliferation, reduced glomeruloid vessels, and blood vessel diameters/perimeters comparable to healthy brain tissue [102]. Notably, even after cediranib discontinuation, no revascularization or rebound angiogenesis was observed, with tumor endothelial cells expressing blood–brain barrier markers [102]. Cediranib also altered rGBM growth patterns, showing lower central tumor cellularity gradually decreasing towards the infiltrating edge, distinct from post-chemoradiation patterns [102]. However, treated tumors exhibited high PDGF-C and c-Met expression, along with significant myeloid cell infiltration, potentially contributing to anti-VEGF resistance [102]. These findings suggest that rGBMs adapt their growth following anti-VEGF therapy, exhibiting decreased cellularity, reduced necrosis, and normalized vasculature without rebound angiogenesis, as well as potential resistance mechanisms.

The NRG/RTOG 0837 trial, a randomized, double-blind, placebo-controlled phase II study, investigated the efficacy of cediranib in newly diagnosed GBM patients [101]. Patients were randomized 2:1 to receive either cediranib (20 mg) or placebo, alongside standard radiation and temozolomide [101]. Of the 158 randomized patients, 137 were eligible and evaluable for the primary endpoint of 6-month progression-free survival [101]. Results showed a significant improvement in 6-month PFS in the cediranib group (46.6%) compared with the placebo group (24.5%), with a *p*-value of 0.005 [101]. However, this improvement in PFS did not translate to a significant difference in overall survival between the two groups [101]. Furthermore, the cediranib group experienced a higher rate of grade 3 or greater adverse events (*p* = 0.02) [101]. While cediranib demonstrated a benefit in short-term disease control, it did not improve overall survival and was associated with increased toxicity [101].

In a study of 31 recurrent GBM patients treated with cediranib, researchers sought early predictive biomarkers for anti-angiogenic therapy response [103]. Using advanced MRI, changes in vascular permeability (*K*trans), microvessel volume, and circulating collagen IV levels were assessed after a single cediranib dose [103]. Changes in these parameters after just one day correlated significantly (*p* < 0.05) with both overall and progression-free survival [103]. A “vascular normalization index”, combining these three parameters, demonstrated a strong correlation with overall survival (ρ = 0.54, *p* = 0.004) and progression-free survival (ρ = 0.6, *p* = 0.001) [103]. This index offers promise as a mechanistic biomarker for predicting survival outcomes in patients receiving anti-VEGF therapy for recurrent GBM, pending validation in randomized clinical trials.

While cediranib’s impact on PFS is encouraging, further research is needed to address its lack of effect on overall survival, manage adverse events, and overcome resistance mechanisms, potentially through combination therapies.

### 3.5. Dovitinib

Dovitinib, a multi-kinase inhibitor targeting FGFR, VEGFR, PDGFRβ, and c-Kit (stem cell factor receptor), has been investigated as a potential treatment for GBM, especially in recurrent cases [77,78]. Its ability to cross the blood–brain barrier and target multiple pathways crucial to GBM development makes it a theoretically attractive option. However, clinical trials have yielded mixed results.

A phase II clinical trial investigated the efficacy of dovitinib in patients with recurrent GBM [78]. The trial comprised two arms: Arm 1 included patients without prior anti-angiogenic therapy, and Arm 2 consisted of patients who had progressed after such therapy [78]. The primary endpoint for Arm 1, 6-month progression-free survival, was a mere 12% ± 6%, indicating that only a small fraction of patients in this group remained progression-free six months post-treatment [78]. Arm 2’s primary endpoint, time to progression, was similar to Arm 1 at a median of 1.8 months, suggesting limited treatment efficacy in both groups [78]. Overall, the study concluded that dovitinib failed to significantly prolong progression-free survival in recurrent GBM patients, regardless of prior anti-angiogenic treatment experience [78]. The majority of patients (70%) experienced disease progression, and 94% had died by the final follow-up, with a median overall survival of 5.6 months [78]. Toxicity was substantial, with 15% of patients experiencing severe (grade 4) toxicities and 67% experiencing moderate (grade 3) toxicities, including lipid abnormalities, elevated liver enzymes, thrombocytopenia, fatigue, and diarrhea [78]. While the study explored biomarker changes, no significant inter-arm differences were observed [78]. However, elevated baseline levels of certain biomarkers, such as BMP 9 (Bone Morphogenetic Protein 9), CD73 (cluster of differentiation 73), and VEGF D, correlated with poorer progression-free survival [78]. Overall, the study indicated limited efficacy for recurrent GBM.

A phase I trial of dovitinib in 12 patients with recurrent GBM (post-radiotherapy and temozolomide) showed that the drug was generally safe and tolerable at 300 mg [77]. Common adverse events were fatigue, elevated liver enzymes, diarrhea, and discomfort. [77]. Severe toxicity (grade 3 or higher) occurred in a minority of patients (16.7%), mainly involving liver enzyme increases and hematotoxicity [77]. However, efficacy was limited; no complete or partial responses were observed [77]. Median progression-free survival was 1.8 months, and median overall survival was 9.5 months [77]. Biomarker analysis suggested a possible reason for the limited efficacy: all tested patients had FGFR3 wild-type but lacked the FGFR3-TACC fusion protein [77]. The trial concluded that dovitinib at 300 mg is safe but not particularly effective in unselected recurrent GBM patients, and future research should explore a personalized approach based on tumor tissue expression of the drug’s target proteins.

Dovitinib’s mechanism of action involves downregulating the stem cell protein Lin28 and its target HMGA2 (High-Mobility Group Protein A2), affecting the STAT3/LIN28/Let-7/HMGA2 regulatory axis in GBM cells [104]. This downregulation reduces tumor sphere formation and enhances temozolomide’s efficacy by impairing DNA (Deoxyribonucleic Acid) repair mechanisms, suggesting a potential combination therapy strategy [104]. However, dovitinib’s clinical use is hampered by significant toxicities, including hepatotoxicity and hematotoxicity [77]. Combining dovitinib with temozolomide has shown increased GBM cell apoptosis and reduced viability in preclinical studies, but this approach also carries increased toxicity risks [104]. While dovitinib holds promise, its clinical efficacy in GBM remains limited, and further research is needed to optimize dosing, patient selection, and combination strategies to improve outcomes and manage toxicity.

### 3.6. Pazopanib

Pazopanib, an anti-angiogenic tyrosine kinase inhibitor of VEGFR, PDGFR, and c-KIT, is also being investigated as a potential GBM treatment.

North American Brain Tumor Consortium Study 06-02, phase II trial, evaluated pazopanib’s efficacy and safety in 35 recurrent GBM patients (median age 53) [105]. Patients received 800 mg/day pazopanib until disease progression or unacceptable toxicity [105]. The primary endpoint, PFS6 (progression free survival at 6 months), was achieved in only one patient (3%) [105]. The median PFS was 12 weeks, and the median OS was 35 weeks [105]. Two patients experienced partial responses, while nine showed some tumor reduction but not enough for partial response classification [105]. Pazopanib was generally well tolerated, with common adverse events including hypertension, fatigue, and elevated liver enzymes [105]. Four patients discontinued treatment due to severe side effects [105]. The study concluded that pazopanib did not significantly prolong PFS, though some biological activity was observed [105]. Compared with other treatments, like bevacizumab, pazopanib’s results were less favorable, suggesting it may not be effective for recurrent GBM at the tested dose.

In the last couple of years, pazopanib has been studied in combination with other drugs. Its effect was associated with primarily grade 1–2 adverse events, including hypertension, increased ALT (alanine transaminase), asthenia, nausea, diarrhea, thrombocytopenia, neutropenia, and anemia [79]. While pazopanib shows potential, its efficacy remains limited, and challenges such as drug delivery and adverse effects need to be addressed. The ongoing PAZOGLIO trial (phase I/II study evaluating pazopanib combined with temozolomide in newly diagnosed GBM patients following the Stupp protocol) and exploration of other combinations, such as with PARP inhibitors, may offer further insights and alternative strategies for GBM treatment [79,106,107].

### 3.7. Ramucirumab

Ramucirumab, a fully humanized monoclonal antibody, specifically targets VEGFR-2, a key receptor in angiogenesis [108]. By binding to VEGFR-2, ramucirumab blocks the binding of VEGF ligands, thereby inhibiting the activation of downstream signaling pathways responsible for endothelial cell proliferation and migration, essential processes for angiogenesis and tumor growth [108,109]. This mechanism aims to restrict tumor blood supply, potentially limiting growth and metastasis [110]. While promising in other cancers, ramucirumab’s efficacy in GBM is still under investigation. The blood–brain barrier poses a significant challenge for drug delivery [111].

Imaging biomarkers VEGF and PDGF receptors were explored to assess ramucirumab’s early biological effects and identify potential therapeutic targets in recurrent GBM [112]. VEGFR-2 and PDGFR were identified as significant targets due to their overexpression in GBM [112]. However, further validation is needed.

A non-randomized phase II clinical trial, NCT00895180, investigated the efficacy of ramucirumab in recurrent GBM patients. In this study, ramucirumab was compared to a monoclonal antibody targeting PDGFR [113]. Results showed that ramucirumab offered marginally improved progression-free survival and overall survival compared to the PDGFR inhibitor [113]. The adverse event profiles of the two treatments were similar [113]. While these findings suggest a potential benefit for ramucirumab in recurrent GBM, the non-randomized nature of the trial limits the strength of the conclusions.

Ramucirumab is generally well-tolerated, but potential adverse events like fatigue and neutropenia warrant consideration [109]. Challenges for ramucirumab’s application in GBM include effective delivery across the blood–brain barrier, potentially marginal benefits compared with existing therapies, and cost-effectiveness [111]. Future research should focus on optimizing delivery methods and identifying specific patient subsets who may derive the most benefit from this therapy.

### 3.8. Sunitinib

Sunitinib is an oral multi-kinase inhibitor targeting several key receptors involved in tumor growth and angiogenesis. Its targets include vascular endothelial growth factor receptors, platelet-derived growth factor receptors, stem cell factor receptors, RET oncogene tyrosine kinase, FMS-like tyrosine kinase 3, and colony-stimulating factor-1 receptor (CSF-1R) [86]. Its intranasal delivery has been explored as a method to bypass the blood–brain barrier and increase drug concentration in the brain, showing promising results in preclinical studies with improved tumor growth reduction and less systemic toxicity compared with oral administration [114]. Intranasal (IN) and oral (OR) administration of sunitinib demonstrated tumor growth reduction in GBM multiforme-bearing rats, confirmed by MRI (magnetic resonance imaging) scans showing decreased tumor size compared with control groups [114]. Both IN and OR delivery effectively inhibited angiogenesis in GBM, indicating sunitinib’s ability to target tumor blood vessel formation [114]. Importantly, IN delivery resulted in less hepatotoxicity than OR administration, suggesting a safer alternative with reduced liver toxicity [114]. These findings support the potential of IN delivery as a non-invasive method to bypass the blood–brain barrier, offering a potentially more effective and safer approach for treating GBM by directly targeting the brain with reduced systemic side effects.

Research by Linde et al. indicates that while intratumoral sunitinib concentrations are higher (reaching a median concentration of 1.9 μmol/L, with a range of 1.0–3.4 μmol/L) than plasma levels (median inhibitory concentration of 5.4 μmol/L, with a range of 3.0–8.5 μmol/L), they remain below the levels required for significant tumor cell growth inhibition in vitro [115]. This suggests that the limited efficacy of sunitinib in GBM may be due to insufficient intratumoral concentrations, highlighting the need for alternative dosing strategies.

The STELLAR trial, investigating high-dose intermittent sunitinib for recurrent GBM, was terminated early due to futility [116]. The trial compared two sunitinib regimens (300 mg once weekly and 700 mg every two weeks) against standard lomustine therapy [116]. Results showed no significant difference in median progression-free survival (mPFS) between the sunitinib groups (1.5 and 1.4 months, respectively) and the lomustine group (1.5 months) [116]. Similarly, median overall survival (mOS) was comparable across groups, ranging from 4.7 to 6.8 months [116]. Only 8% of patients in both sunitinib groups were progression-free at six months, compared with 15% in the lomustine group [116]. Both treatments were generally well tolerated, with maximal grade 3 toxicities observed in 8% of sunitinib patients and 15% of lomustine patients [116]. The study concluded that high-dose intermittent sunitinib did not improve outcomes compared with lomustine, highlighting the need for more effective recurrent GBM treatments.

Overall, while alternative delivery methods and combination therapies offer potential avenues for enhancing sunitinib’s effectiveness, further research is needed to overcome the challenges posed by the blood–brain barrier and optimize treatment strategies for GBM patients.

## 4. Combination Therapy

Combination therapy in GBM angiogenesis utilizes multiple therapeutic agents to target the complex interplay of mechanisms driving tumor growth and vascularization. This approach seeks to improve treatment efficacy by addressing the shortcomings of single-agent therapies, such as drug resistance and incomplete suppression of angiogenic pathways. Various studies have investigated different drug combinations to inhibit angiogenesis in GBM, concentrating on key signaling pathways and molecular mechanisms crucial for tumor progression (Table 3).

## 5. Resistance to Therapy

**Figure 2:**
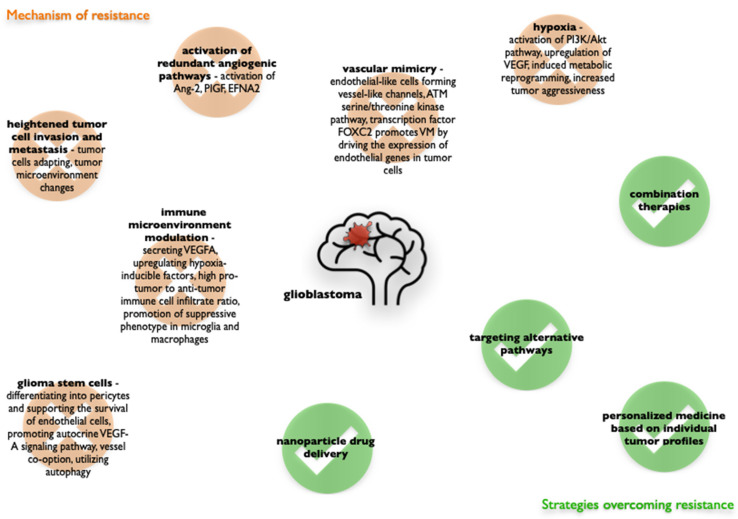
Glioblastoma—mechanisms of resistance to anti-angiogenic therapy and strategies to overcome resistance.

Resistance to anti-angiogenic therapy in GBM presents a significant hurdle in treatment (Figure 2). GBM employs several mechanisms to circumvent therapies designed to inhibit blood vessel growth.

### 5.1. Mechanisms of Resistance—Activation of Redundant Angiogenic Pathways

GBM exhibits a remarkable ability to adapt to anti-angiogenic therapies by activating alternative pathways for blood vessel formation [134,135]. When common therapies like bevacizumab inhibit vascular endothelial growth factor, GBM can upregulate other pro-angiogenic factors [134,135]. Specifically, angiopoietin-2 (Ang-2), placental growth factor (PlGF), and ephrin A2 (EFNA2) have been observed at increased levels in GBM tissues resistant to bevacizumab [134]. This activation of alternative angiogenic pathways allows GBM to continue developing new blood vessels, even when VEGF signaling is suppressed, contributing to treatment resistance and continued tumor growth [134].

### 5.2. Mechanisms of Resistance—Hypoxia

Hypoxia, a characteristic of the tumor microenvironment exacerbated by anti-angiogenic therapies, plays a significant role in GBM resistance [135,136,137]. Under low oxygen conditions, tumor cells activate survival pathways, notably the PI3K/Akt pathway, enabling continued growth and division [135,138]. Hypoxia-inducible factors are stabilized and activated, promoting angiogenesis by upregulating pro-angiogenic factors like VEGF, counteracting the effects of anti-angiogenic therapies [135,137]. Additionally, hypoxia induces metabolic reprogramming, allowing the tumor to utilize alternative energy sources and maintain growth despite a reduced blood supply [135,137]. Furthermore, hypoxia can increase tumor aggressiveness, enhancing invasive and metastatic potential, thereby complicating treatment strategies [135,137]

### 5.3. Mechanisms of Resistance—Heightened Tumor Cell Invasion and Metastasis

When anti-angiogenic therapies restrict the tumor’s blood supply, GBM cells become more invasive, seeking out new vessels and spreading aggressively within the brain [135,139]. While GBM typically doesn’t metastasize outside the central nervous system, this heightened invasiveness leads to more widespread infiltration, complicating treatment [135]. Tumor cells adapt by altering cell adhesion properties and expressing enzymes that degrade the extracellular matrix, facilitating movement through tissue barriers [135]. Furthermore, the tumor microenvironment changes in response to therapy, creating conditions that favor invasion and metastasis, including alterations in the extracellular matrix and interactions with other cell types [135].

### 5.4. Mechanisms of Resistance—Vascular Mimicry

Vascular mimicry (VM) is a process where tumor cells form vessel-like channels independent of traditional angiogenesis, providing an alternative blood supply [135,140]. In GBM, these channels, formed by tumor cells rather than endothelial cells, allow the tumor to receive nutrients and oxygen, especially when anti-angiogenic therapies are employed [135]. This process represents a key resistance mechanism, enabling tumor growth and survival despite therapies targeting traditional blood vessel formation [135]. Glioma stem-like cells contribute to vascular mimicry in GBM by transdifferentiating into endothelial-like cells, forming vessel-like channels that bypass traditional angiogenesis [140]. This process is facilitated by pathways such as ATM serine/threonine kinase, which is also implicated in chemoradiotherapy resistance [140]. Furthermore, the transcription factor FOXC2 promotes VM by driving the expression of endothelial genes in tumor cells, a process amplified by hypoxia within the tumor microenvironment [141]. This hypoxia-induced VM formation further enhances resistance to anti-angiogenic therapies [141].

### 5.5. Mechanisms of Resistance—Glioma Stem Cells

Glioma stem cells play a critical role in GBM’s resistance to anti-angiogenic therapies [135,142]. GSCs can differentiate into pericytes, supporting the survival of endothelial cells crucial for maintaining tumor blood vessels [142]. Furthermore, GSCs promote an autocrine VEGF-A signaling pathway, which helps sustain the tumor’s blood supply, even when targeted by therapies [142]. GSCs are also involved in vessel co-option, allowing the tumor to hijack existing blood vessels and bypass the need for new vessel formation [140,142]. Under hypoxic conditions, GSCs utilize autophagy to survive, adapting to the stress induced by treatment [142]. These combined mechanisms demonstrate how GSCs contribute significantly to GBM’s resistance to therapies aimed at disrupting its blood supply, making them a key target for future treatment strategies.

### 5.6. Mechanisms of Resistance—Immune Microenvironment Modulation

GBM tumor microenvironment is a complex interplay of cellular and non-cellular components, including glioma cells, glioma stem cells, immune cells (macrophages, microglia), and the extracellular matrix [143,144]. These components dynamically interact, influencing tumor growth, progression, and therapeutic resistance [135,143,144]. Tumor-associated macrophages and myeloid-derived suppressor cells contribute to the immunosuppressive TME (tumor microenvironment) by secreting pro-angiogenic factors like VEGFA, promoting angiogenesis and tumor growth, thereby hindering anti-angiogenic therapies [145,146]. Hypoxia within the TME further exacerbates immunosuppression by upregulating hypoxia-inducible factors, which polarize myeloid cells towards a suppressive phenotype, inhibiting effective immune responses [147]. GBM develops resistance to anti-angiogenic therapies through redundant pro-angiogenic pathways, increased tumor cell invasion, and the formation of vasculogenic mimicry channels, which bypass traditional blood vessels [135,145]. The “cold” immune environment, characterized by a high pro-tumor to anti-tumor immune cell infiltrate ratio, further contributes to resistance, particularly against immunotherapy [148]. Granulocyte-rich environments exacerbate this immunosuppression by promoting a suppressive phenotype in microglia and macrophages, hindering therapies targeting VEGF [146].

Overcoming resistance to anti-angiogenic therapy in GBM is a significant challenge due to the tumor’s complex biology and adaptive mechanisms. While initially promising, therapies targeting VEGF often encounter resistance, limiting long-term efficacy. Several strategies aim to address this, including combination therapies (e.g., bevacizumab with SB431542), targeting alternative pathways (e.g., MNK-eIF4E axis with tomivosertib), personalized medicine based on individual tumor profiles, and nanoparticle drug delivery for improved precision (Figure 2) [135,149,150]. Emerging perspectives emphasize the importance of multi-target approaches, including natural products and novel drug combinations, as well as a deeper understanding of the tumor microenvironment, particularly immune modulation and macrophage activity, to develop more effective and sustainable strategies to overcome resistance in GBM [151,152].

## 6. Conclusions

Anti-angiogenic therapy initially held promise for GBM due to the tumor’s highly vascular nature. However, resistance mechanisms limit long-term efficacy and overall survival benefits. GBM tumors frequently activate alternative pro-angiogenic pathways, bypassing the effects of agents like bevacizumab. Hypoxia induced by anti-angiogenic therapy paradoxically promotes tumor invasion and metastasis. The tumor microenvironment’s immune components are also modulated, potentially reducing treatment effectiveness. While anti-angiogenic therapy improves progression-free survival, it does not significantly impact overall survival. This suggests that while disease progression may be delayed, the ultimate trajectory remains unchanged. Furthermore, combining anti-angiogenic therapy with chemoradiotherapy increases adverse events, particularly hematologic ones. Current research focuses on combination therapies with immunotherapy or personalized medicine, alternative dosing regimens, and next-generation anti-angiogenic agents like small interfering RNAs and nanoparticles to overcome resistance and improve outcomes.

## Figures and Tables

**Table 1:** Adverse effects of anti-angiogenic agents.

Anti-Angiogenic Agent	Protein Targets	Adverse Effects	References
aflibercept	VEGF-A, VEGF-B, PlGF-1, PlGF-2	hypertension thromboembolic events (deep vein thrombosis and pulmonary embolism) hemorrhagic complications proteinuria fatigue gastrointestinal and nasal septum perforation	[60,61,62,63,64,65]
axitinib	VEGFRs, c-Kit, PDGFR	fatigue diarrhea hypertension oral hyperesthesia dysphonia changes in voice	[66]
bevacizumab	VEGF-A	hypertension proteinuria fatigue neutropenia (more common when bevacizumab is combined with other therapies) increased risk of thromboembolic events (when combined with chemotherapy or anticoagulants) hemorrhagic events gastrointestinal perforation neurocognitive dysfunction (potentially impacting memory and information processing)	[67,68,69,70,71,72,73,74,75]
cediranib	VEGFRs, c-Kit	fatigue diarrhea hypertension	[76]
dovitinib	FGFRs, VEGFRs, PDGFR	severe fatigue diarrhea liver enzyme elevation	[77,78]
pazopanib	VEGFRs, c-Kit, PDGFR	thrombocytopenia neutropenia hypertension elevated ALT levels asthenia nausea diarrhea anemia	[79]
ramucirumab	VEGFR2	hypertension bleeding febrile neutropenia neutropenia thrombocytopenia nephrotic syndrome gastrointestinal perforation sporadic angioma transient vocal fold lesions hoarseness	[80,81,82,83,84]
sunitinib	VEGFRs, c-Kit, PDGFR, Flt-3, RET, CSF-1R	fatigue neutropenia thrombocytopenia diarrhea nausea hand-foot syndrome neurological deterioration central nervous system hemorrhage	[85,86,87,88]

**Table 2:** Anti-angiogenic agents clinical trials.

**Anti-Angiogenic Agent**	**Clinical Trial Number**
aflibercept	NCT00650923
axitinib	NCT01562197, NCT03291314, NCT01508117
bevacizumab	NCT01102595, NCT02285959, NCT02511405, NCT01091792, NCT01349660, NCT05476341, NCT05871021, NCT01564914, NCT00805961, NCT02342379, NCT02669173, NCT00800917, NCT01269853, NCT01308684, NCT03452579, NCT01933815, NCT02337491, NCT02047214, NCT00345163, NCT02698280, NCT05811793, NCT01939574, NCT01115491, NCT01782976, NCT05502991, NCT00501891, NCT01811498, NCT00892177, NCT01632228, NCT01435395, NCT02060955, NCT03661723, NCT00817284, NCT00762255, NCT00590681, NCT01443676, NCT01474239, NCT00904852, NCT01209442, NCT03149003, NCT00921167, NCT05540275, NCT05191784, NCT04952571, NCT02898012, NCT02761070, NCT06061809, NCT00671970, NCT00612430, NCT01067469, NCT02157103, NCT00967330, NCT00525525, NCT01331616, NCT02017717, NCT00943826, NCT00883298, NCT01266031, NCT02743078, NCT00613028, NCT01113398, NCT03743662, NCT02120287, NCT05638451, NCT05201326, NCT05118776, NCT00611325, NCT02974621, NCT02386826, NCT01860638, NCT00735436, NCT02768389, NCT01526837, NCT00979017, NCT00939991, NCT02841332, NCT04446416, NCT05284643, NCT01663012, NCT06329570, NCT02663271, NCT00612339, NCT01609790, NCT04681677, NCT01186406, NCT01814813, NCT04074785, NCT01730950, NCT01086345, NCT03631836, NCT01618747, NCT05271240, NCT03573986, NCT03532295, NCT00968240, NCT00463073, NCT02330562, NCT01149850, NCT03890952, NCT04974983, NCT01987830, NCT00884741, NCT06496971, NCT01582152, NCT00586508, NCT02833701, NCT00597402, NCT01413438, NCT03856099, NCT01925573, NCT00621686, NCT02754362, NCT01004874, NCT03149575, NCT01022918, NCT04566185, NCT01894061, NCT00433381, NCT01290939, NCT01740258, NCT01931098, NCT00906516, NCT06160206, NCT00720356, NCT01648348, NCT03144167, NCT05611645, NCT00337207, NCT02343549, NCT02142803, NCT00973557, NCT04143425, NCT02348255, NCT00458731, NCT01478321, NCT01392209, NCT00667394, NCT03623347, NCT00795665, NCT01810744, NCT00879437, NCT01013285, NCT01339039, NCT01189240, NCT00782756, NCT00352521
cediranib	NCT00777153, NCT02974621, NCT01310855, NCT00503204, NCT00979862, NCT00458731, NCT01062425, NCT00662506, NCT01131234
dovitinib	NCT01972750, NCT01753713
pazopanib	NCT02331498, NCT01931098, NCT00459381, NCT01392352, NCT00350727
ramucirumab	NCT00895180
sunitinib	NCT00535379, NCT00611728, NCT00606008, NCT03025893, NCT01100177, NCT02928575, NCT00864864, NCT00923117, NCT00499473, NCT01122888

**Table 3:** Anti-angiogenic agents—combination therapy.

Anti-Angiogenic Agent	Combination Agent	Study Description	References
aflibercept	radiation therapy, temozolomide	Study Design: Phase I, 3+3 dose-escalation, three arms with different aflibercept/temozolomide combinations. Patient Population: 59 patients with newly diagnosed high-grade gliomas. Study Objective: Determine the maximum tolerated dose of aflibercept. MTD (Maximum tolerated dose): 4 mg/kg every two weeks for all arms. Dose-Limiting Toxicities: deep vein thrombosis, neutropenia, thrombotic microangiopathy, rash, thrombocytopenia Reasons for Treatment Discontinuation: Primarily disease progression and toxicities. Treatment Duration: Median of five aflibercept cycles.	[91]
axitinib	avelumab	Study Design: Phase II, open-label, single-center, stratified. Patient Population: Recurrent GBM patients, stratified by corticosteroid use. Intervention: Cohort 1: concurrent axitinib and avelumab. Cohort 2: axitinib monotherapy initially, avelumab added after corticosteroid reduction. Primary Endpoint: 6-month progression-free survival. Results: Cohort 1: 22.2% 6-month PFS, 26.6 weeks median overall survival. Cohort 2: 18.5% 6-month PFS, 18.0 weeks median OS. Safety: Generally well-tolerated. Common adverse events included dysphonia, lymphopenia, hypertension, and diarrhea. Conclusion: Did not meet efficacy threshold for further study in an unselected recurrent GBM population.	[117]
axitinib	lomustine	Study Design: Randomized phase II trial. Patient Population: Recurrent GBM (rGB). Interventions: Axitinib monotherapy vs. axitinib plus lomustine. Primary Endpoint: 6-month progression-free survival (6mPFS). Results: 6mPFS: 26% AXI, 17% AXILOM (no significant benefit for combination). Median overall survival (mOS): 29 weeks AXI, 27.4 weeks AXILOM (similar). Best overall response rate: 38% AXILOM, 28% AXI (higher in combination, but with increased toxicity). Safety: Increased grade 3/4 neutropenia and thrombocytopenia in the AXILOM arm. Crossover: AXI arm patients could crossover to AXILOM upon progression.	[118]
bevacizumab	irinotecan	Study Design: Randomized Phase II trial. Patient Population: 846 patients with recurrent GBM following surgery/biopsy and chemoradiotherapy. Interventions: Bevacizumab monotherapy vs. bevacizumab plus irinotecan. Primary Outcome: Overall survival from initial surgery. Results: No significant difference in overall survival (22.6 months for bevacizumab vs. 20.44 months for bevacizumab + irinotecan). B+I group received a significantly higher median number of bevacizumab prescriptions (5 vs. 3). Factors associated with decreased survival: Male sex, older age, biopsy (vs. resection), and higher number of radiotherapy cycles. Conclusion: Bevacizumab monotherapy is a reasonable option considering the lack of added survival benefit with irinotecan and the potential for increased toxicity with combination therapy.	[119]
bevacizumab	temozolomide, radiotherapy	Study Design: Systematic review and meta-analysis of six studies. Intervention: Addition of bevacizumab to temozolomide and radiotherapy for GBM multiforme. Outcomes: Overall survival and progression-free survival. Results: No significant improvement in OS or PFS with bevacizumab. Pooled odds ratio for OS: 0.843 (95% CI 0.615–1.156, *p* = 0.290). Pooled odds ratio for PFS: 0.829 (95% CI 0.561–1.224, *p* = 0.346). Conclusion: Bevacizumab does not offer additional benefit in this setting.	[120,121]
bevacizumab	re-irradiation	Context: Recurrent GBM has a poor prognosis and lacks a standard treatment approach. Treatment Strategy: Re-irradiation combined with bevacizumab is frequently used. Potential Advantages: This combination may offer acceptable toxicity, especially with appropriate fractionation, making it a potentially safer option. Current Status: While various re-irradiation and bevacizumab regimens have been explored, further research is crucial to optimize treatment protocols. Challenges and Future Directions: Further research is needed to address existing challenges and improve treatment efficacy.	[122]
bevacizumab	Gamma Knife	Study Design: Comparison of three treatment groups for recurrent GBM (rGBM) at first recurrence. Treatment Groups: Bevacizumab only, Gamma Knife radiosurgery only, Combined bevacizumab and Gamma Knife Outcome Measures: Post-recurrence progression-free survival and overall survival. Key Findings: The combined treatment group showed significantly improved PFS (7.7 months) and OS (11.5 months). These improvements were statistically significant compared to either treatment alone (*p* = 0.015 for total PFS, *p* = 0.0050 for total OS, *p* = 0.018 for post-recurrence PFS, and *p* = 0.0082 for post-recurrence OS). Conclusion: Concurrent bevacizumab and Gamma Knife radiosurgery appear to enhance survival in recurrent GBM.	[123]
bevacizumab	irinotecan	Study Design: Phase II trial in 167 patients with recurrent GBM. Treatment Arms: Bevacizumab monotherapy vs. bevacizumab plus irinotecan. Outcomes: 6-month progression-free survival: 42.6% (bevacizumab) vs. 50.3% (combination). Objective response rate: 28.2% (bevacizumab) vs. 37.8% (combination). Median overall survival: 9.2 months (bevacizumab) vs. 8.7 months (combination). Safety: Grade 3 or higher adverse events: 46.4% (bevacizumab) vs. 65.8% (combination). Conclusion: While both treatments showed activity, adding irinotecan did not significantly improve OS despite increasing PFS and ORR.	[70]
bevacizumab	irinotecan	Study Design: Analysis of outcomes in 846 recurrent GBM patients. Treatment Groups: Bevacizumab monotherapy (BEV, n = 450), Bevacizumab plus irinotecan (B+I, n = 396) Demographics: The BEV group was older and had a higher proportion of females. Primary Outcome: Overall survival Results: No significant difference in OS between the two treatment groups. Prognostic Factors: Younger age, female gender, and prior surgery (vs. biopsy) were associated with better prognosis. Conclusion: Bevacizumab monotherapy is a reasonable treatment option, offering similar efficacy to combination therapy with potentially less toxicity.	[124]
bevacizumab	sorafenib	Objective: To evaluate the efficacy and safety of combining bevacizumab and sorafenib, targeting vertical VEGF signaling blockade, in recurrent GBM. Study Design: Phase II trial with two sorafenib dosing groups due to initial toxicity concerns. Results: No significant improvement in outcomes compared to historical bevacizumab monotherapy data. Objective response rate: 18.5%. 6-month progression-free survival: 20.4%. Median overall survival: 5.6 months. Biomarkers: Associations found between SNPs in VEGF and VEGFR2, baseline stromal cell-derived factor-1 levels, and PFS6. Circulating endothelial cells and ADC-L identified as potential biomarkers. Toxicity: Significant toxicity observed, leading to dose adjustments. Conclusion: While the combination did not improve efficacy, the study provided valuable insights into potential biomarkers and highlighted the challenges of combination anti-VEGF therapies in GBM.	[125]
bevacizumab	lomustine	Study Design: Randomized phase II trial investigating bevacizumab plus CCNU (lomustine) in recurrent GBM. Molecular Profiling: Gene expression profiling was used to classify tumors. Key Finding: Patients with the IGS-18/classical subtype experienced significantly improved progression-free survival and a trend toward better overall survival with the combination therapy. Subtype-Specific Benefit: Other molecular subtypes did not show similar benefits. Conclusion: Tumor classification is important for predicting treatment response to bevacizumab and CCNU in recurrent GBM. The combination appears most effective in the IGS-18/classical subtype.	[126]
bevacizumab	fotemustine, irinotecan, temozolomide, lomustine	Study Population: 160 patients with recurrent GBM treated with bevacizumab plus chemotherapy. Treatment Groups: Bevacizumab + fotemustine (n = 100), Bevacizumab + another cytotoxic agent (irinotecan, temozolomide, or lomustine; n = 62) Outcomes: Median progression-free survival: 4.47 months (entire cohort). Median overall survival: 9 months (entire cohort); 7.3 months (fotemustine group); 19.9 months (other cytotoxic agents group). 3-month disease control rate: 51%. Prognostic Factors: Baseline steroid use and low Karnofsky performance status associated with poorer survival. Adverse Events: Grade 3–4 adverse events: 21.9% of patients (no difference between groups). Grade 5 adverse events: 7 patients in the fotemustine group. Conclusion: Bevacizumab plus fotemustine was less effective than bevacizumab combined with other cytotoxic agents in this real-world setting.	[127]
bevacizumab	fotemustine	Patients: Forty-two recurrent GBM patients (16 women, 25 men; median age 52). Most (85.4%) had a good performance status (ECOG 0-1). Prior Treatments: All patients received prior radiotherapy and temozolomide. A majority had prior tumor resection (total/subtotal or partial). About 36% experienced relapse within 3 months of initial treatment. Relapse location was unifocal in 61% and multifocal in 39%. Treatment Response: Stable disease: 46.3%. Partial response: 24.4%. Complete response: 2.4%. Survival: Median progression-free survival: 6 months. Median overall survival: 7 months. Toxicity: Grade 3–4 toxicity occurred in 22% of patients, primarily hematologic adverse events. Comparison to Other Studies: Results were comparable to other studies using this combination, though with slightly higher PFS but lower OS than a previous phase II study, potentially due to more rapid progressors in this cohort.	[128]
bevacizumab	vorinostat	Trial Design: Compared bevacizumab plus vorinostat versus bevacizumab alone in patients with recurrent GBM. Outcomes: No significant difference in outcomes between the two groups. Median progression-free survival: 3.7 months (combination) vs. 3.9 months (bevacizumab monotherapy). Median overall survival: 7.8 months (combination) vs. 9.3 months (bevacizumab monotherapy). No improvement in clinical benefit or quality of life. Safety: Grade ≥ 3 toxicities were observed, including hypertension, neurological changes, anorexia, infections, wound dehiscence, thromboembolic events, and colonic perforation. However, the combination was generally well-tolerated. Conclusion: The addition of vorinostat to bevacizumab did not offer a significant advantage over bevacizumab monotherapy in recurrent GBM. The trial successfully demonstrated the feasibility of a Bayesian adaptive design in this setting.	[129]
bevacizumab	temozolomide	Study Focus: Evaluated the impact of adding bevacizumab to temozolomide in newly diagnosed IDH-wildtype GBM. Compared outcomes in three eras: pre-TMZ, TMZ alone, and TMZ + BEV. Overall Survival: Median OS increased with the introduction of TMZ: 14.6 months (pre-TMZ) to 14.9 months. Further OS improvement with BEV addition: 22.1 months (TMZ + BEV). Prognostic Factors: Extent of resection and MGMT methylation status were significant prognostic factors in the TMZ era but not in the TMZ + BEV era. Subgroup Analysis: TMZ improved OS in MGMT-methylated patients, although not significantly (*p* = 0.13). BEV significantly improved OS in MGMT-unmethylated patients (*p* = 0.04). Conclusion: First-line BEV complements TMZ and may extend survival, particularly in patients without MGMT methylation. This supports personalized treatment strategies based on prognostic factors.	[130]
cediranib	radiotherapy, temozolomide	Trial Design: A randomized, double-blind, placebo-controlled phase II study evaluating cediranib added to standard radiotherapy and temozolomide in newly diagnosed GBM. Key Finding: Cediranib significantly improved 6-month progression-free survival (46.6% vs. 24.5%, *p* = 0.005). Overall Survival: Despite the improvement in progression-free survival, there was no significant overall survival benefit with the addition of cediranib. Adverse Events: Cediranib was associated with a higher rate of severe adverse events.	[101]
cediranib	olaparib	Trial Design: Compared cediranib/olaparib combination therapy to bevacizumab monotherapy in 70 recurrent GBM patients. Treatment Arms: Cediranib 30 mg daily + olaparib 200 mg twice daily. Bevacizumab 10 mg/kg every two weeks. Outcomes: No significant difference in survival outcomes between the two groups. Median progression-free survival: 118 days (cediranib/olaparib) vs. 92 days (bevacizumab). Median overall survival: 269.5 days (cediranib/olaparib) vs. 192 days (bevacizumab) Genomic Analysis: Whole exome sequencing was performed on a subset of patients (details not provided). Conclusion: The combination of cediranib and olaparib did not demonstrate a significant survival benefit compared to bevacizumab alone in recurrent GBM.	[131]
pazopanib	temozolomide	Trial Design: A phase I/II study evaluating pazopanib combined with temozolomide during maintenance therapy for resected GBM. Phase I: 20 patients enrolled across four pazopanib dose levels (200 mg, 400 mg, 600 mg, 800 mg). One dose-limiting toxicity observed at 600 mg. Two DLTs observed at 800 mg (primarily thrombocytopenia and hypertension). Most adverse events were grade 1–2. Recommended Phase II Dose (RP2D): 600 mg pazopanib with temozolomide, although frequent dose adjustments were required. Conclusion: The combination was deemed feasible, and the phase II portion of the trial is ongoing.	[79]
pazopanib	lapatinib	Trial Design: A phase I/II trial investigating the combination of pazopanib and lapatinib for malignant glioma. The trial was terminated early during phase II due to limited efficacy. Phase II Results: Low progression-free survival rates at 6 months: 0% in patients positive for both PTEN and EGFRvIII. 15% in patients negative for PTEN and/or EGFRvIII. Efficacy: Limited overall anti-tumor activity was observed despite some patients experiencing partial response or stable disease. Phase I Results: A safe dosage regimen was determined in phase I, but the maximum tolerated dose was not reached. Drug Interactions: Concomitant use of enzyme-inducing anticonvulsants reduced drug exposure, potentially impacting efficacy. Conclusion: The combination therapy showed limited efficacy, possibly due to subtherapeutic lapatinib exposure. The study highlights the need to explore alternative drug delivery methods, such as intratumoral delivery, in future trials.	[132]
sunitinib	temozolomide radiotherapy	Trial Design: Phase II trial evaluating sunitinib in combination with temozolomide and radiotherapy in newly diagnosed GBM patients with unmethylated MGMT. Key Outcomes: Median progression-free survival: 7.15 months. Median overall survival: 15 months Positive Prognostic Factors for Overall Survival: Receiving more than three cycles of adjuvant TMZ. Undergoing surgery at progression. Neutrophil-to-lymphocyte ratio ≤ 6 Negative Prognostic Factor for Overall Survival: Age over 65 Adverse Events: Grade 3 thrombocytopenia, neutropenia, and thromboembolic events were observed; no grade 5 events occurred. Conclusion: Suggests potential benefits for this combination therapy, warranting further investigation.	[133]

## Data Availability

No new data were created or analyzed in this study. Data sharing is not applicable to this article.
